# Sulfurization engineering of single-zone CVD vertical and horizontal MoS_2_ on p-GaN heterostructures for self-powered UV photodetectors†

**DOI:** 10.1039/d2na00756h

**Published:** 2023-01-10

**Authors:** Nur 'Adnin Akmar Zulkifli, Nor Hilmi Zahir, Atiena Husna Abdullah Ripain, Suhana Mohd Said, Rozalina Zakaria

**Affiliations:** a Photonic Research Centre, University Malaya 50603 Kuala Lumpur Malaysia rozalina@um.edu.my; b Low Dimensional Material Research Center (LDMRC), Physics Dept. Faculty of Science, University Malaya 50603 Kuala Lumpur Malaysia; c Department of Electrical Engineering, Faculty of Engineering, University of Malaya 50603 Kuala Lumpur Malaysia

## Abstract

Molybdenum disulfide (MoS_2_) has been attracting considerable attention due to its excellent electrical and optical properties. We successfully grew high-quality, large-area and uniform few-layer (FL)-MoS_2_ on p-doped gallium nitride (p-GaN) using a simplified sulfurization technique by the single-zone CVD of a Mo seed layer *via* E-beam evaporation. Tuning the sulfurization parameters, namely temperature and duration, has been discovered to be an effective strategy for improving MoS_2_ orientation (horizontally aligned and vertically aligned) and quality, which affects photodetector (PD) performance. The increase in the sulfurization temperature to 850 °C results in improved structural quality and crystallite size. However, a prolonged sulfurization duration of 60 minutes caused the degradation of the film quality. The close lattice match between p-GaN and MoS_2_ contributes to the excellent quality growth of deposited MoS_2_. Following this, an n-MoS_2_/p-GaN heterostructure PD was successfully built by a MoS_2_ position-selectivity method. We report a highly sensitive and self-powered GaN/MoS_2_ p–n heterojunction PD with a relatively high responsivity of 14.3 A W^−1^, a high specific detectivity of 1.12 × 10^13^ Jones, and a fast response speed of 8.3/13.4 μs (20 kHz) under a UV light of 355 nm at zero-bias voltage. Our PD exhibits superior performance to that of the previously reported MoS_2_/GaN p–n PD. Our findings suggest a more efficient and straightforward approach to building high-performance self-powered UV PDs.

## Abstract 2

Schematic diagram of the n-MoS_2_/p-GaN heterojunction photodetector fabricated and light intensity-dependent of responsivity and specific detectivity of the 30 minutes device.

## Graphical abstract

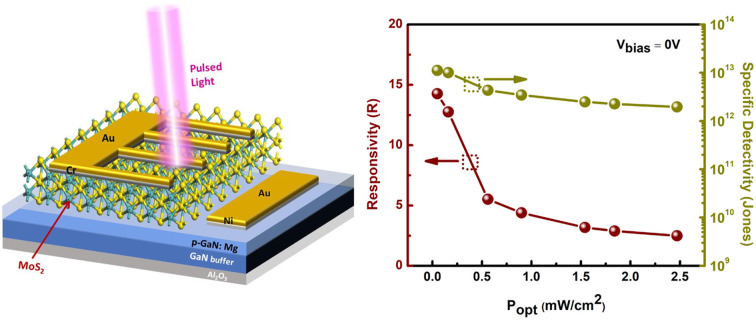

## Introduction

In recent years, driven by the exceptional properties of graphenes and fascinating novel devices offered by 2-dimensional layered materials, researchers have aggressively shifted their focus to the finding of a 2D material with finite bandgap energies. Despite graphene's fast absorption and broad-spectrum light absorption, its low photocarrier lifetime and weakly visible spectrum irradiation absorption hamper its application in photodetectors. Two-dimensional dichalcogenides (2D TMDs) show potential application, particularly in photodetection, due to their remarkable electronic and optical properties including tunable bandgap and ultrahigh broadband light absorption because of different numbers of layers,^1^ large electronic density of states resulting in high optical absorption and ultrafast charge transfer, strong light–matter interactions,^2^ high charge carrier mobility,^3,4^ and the ability to create van der Waals (vdW) heterostructures with atomically sharp interfaces. van der Waals heterostructures with distinct layers of 2D TMDs have been fabricated for enhanced performance in optoelectronic applications. However, due to several limitations in fabricating such heterostructures,^5,6^ researchers began to benefit from the 2D/3D heterostructure for real device applications due to the enhancement in its photoresponse.

Unlike the p–n homojunction, the p–n heterojunction is created by combining two distinct semiconductor materials with varying bandgaps and properties, which could significantly enhance semiconductor device's flexibility. It is also an effective way to improve the separation efficiency of photoexcited electron–hole pairs by using an electric field built into the device.^7,8^ Additionally, a p–n heterostructure with a photovoltaic effect can be used to build an independently powered device that runs without an external power source. The seamless compatibility of Si-based substrates such as amorphous SiO_2_ has been commonly used for MoS_2_ growth for photodetector application. However, these photodetectors deteriorated due to high gate voltage and low photoresponsivity.^9,10^

Recent research has discovered that single crystals such as quartz, mica, and sapphire can also be incorporated to grow high-quality MoS_2_.^11^ The advantage of adopting single-crystal substrates is their excellent temperature stability, chemical inertness, and distinctive hexagonal surface arrangement. Their atomically flat surface may also aid precursor migration during CVD, enhancing the thickness homogeneity of the MoS_2_ layers produced.^12^ Additionally, the close in-plane lattice match of MoS_2_ and GaN with only 0.7 percent lattice mismatch^13^ and similar hexagonal arrangement^14^ have gained the interest of researchers to explore this particular heterostructure as a promising platform for electronic devices.

Moreover, GaN is expected to be an excellent candidate for UV photodetectors (PDs) due to its wide direct bandgap (3.4 eV), exceptional radiation hardness, and high thermal stability.^15^ The MoS_2_/GaN heterojunction has been recently reported as a promising platform for electronic devices.^16,17^ Despite this, the MoS_2_/GaN p–n heterojunction has received little attention due to the difficulties associated with GaN p-type doping.

The synthesis of hexagonal 2D TMDCs for the growth of high-quality ultrathin films with layer controllability and large-area uniformity is extensively performed for various device applications. Primary techniques to obtain atomically thin MoS_2_ layers can be classified into two types. Top-down fabrication techniques such as scotch-tape-based cleavage, chemical etching, and laser thinning involve the exfoliation of bulk crystals down to a micrometer-sized layer. The bottom-up technique involves deposition of molybdenum precursors and sulfur (S) on a substrate *via* techniques such as chemical vapor deposition (CVD), physical vapor deposition (PVD) and atomic layer deposition (ALD) of MoS_2_ layers. Although mechanical cleavage of MoS_2_ has been of interest due to its high electrical performance quality, the inability to control the number of layers is a major drawback of this method.

However, the well-known CVD involving a vapor-phase reaction between MoO_3_ and S powders is constrained by lateral scaling and thickness control. To solve this issue, recent research has opted for thermal vapour sulfurization (TVS) CVD, in which the source materials (MoO_3_ and Mo precursor) are first pre-deposited on a substrate *via* electron-beam (E-beam) evaporation or sputtering.^18^ This method is favorable due to its ability to control the thickness of MoS_2_ based on the initial thickness of the Mo-based film^19^ and the particular growth area of MoS_2_.^20^ The effects of both sulfurization temperature and pressure on Mo-based films deposited by magnetron sputtering and ALD have also been investigated^19,21,22^ in a two-zone and three-zone furnace. To note, no previous research has thoroughly explained the systematic effect of both temperature and duration on the 2D-MoS_2_ growth in a single-zone furnace, and plasma bombardment is known to cause surface damage to p-type GaN.^23^ Furthermore, many works concentrated primarily on MoS_2_ synthesis, but their practical applications as devices were overlooked.

The current work aims to investigate the effect of sulfurization temperature (650–850 °C) and duration (15–60 minutes) on the growth of thin (6–7 layers) MoS_2_ films obtained by E-beam deposition of a Mo seed layer on a p-GaN substrate. Various characterization techniques including XRR, Raman, HR-XRD and FE-SEM analysis were used to analyse the structural quality and morphological properties of grown MoS_2_ nanosheets. Furthermore, to thoroughly compare the performance of deposited MoS_2_, n-MoS_2_/p-GaN heterostructure PDs were built, and the photoelectric performance was evaluated and carefully discussed.

## Experimental section

### Growth of p-GaN

A 1.5 μm p-GaN film (carrier concentration: 1.3 × 10^18^ cm^3^) was deposited on sapphire by metal oxide chemical vapor deposition (MOCVD). Trimethylgallium (TMG) and ammonia (NH_3_) were used as sources for the GaN film formation, while bis(cyclopentadienyl)magnesium (Cp_2_Mg) was used as a source for the p-dopant. The p-GaN layer was pre-annealed at 650 °C for 15 minutes to activate it. To remove organic contaminants, the samples were ultrasonically cleaned in acetone, isopropanol, and de-ionized water (DI-Water) for 5 minutes each. The samples were immersed in a 1 : 3 HCl : H_2_O solution for 30 seconds to remove the native oxide layer from the surface. The samples were rinsed with DI water and then dried using a nitrogen gun before Molybdenum (Mo) deposition.

### Synthesis of large-area MoS_2_ thin films

In our two-step method, a 2 nm Mo seed layer (99.95%, Kurt J. Lesker) was initially deposited at room temperature on c-plane p-GaN/sapphire (1 cm × 1 cm) substrates *via* electron-beam evaporation (EB43-T) at a deposition rate of 0.1 Å s^−1^. The vacuum chamber was evacuated to 2.0 × 10^−6^ Pa before the coating process started. CVD growth was carried out at atmospheric pressure with high-purity N (99.99% purity) as a carrier gas in a single-zone temperature tube furnace. Then, the as-deposited Mo layer was set on a ceramic crucible, inserted into a 4-inch diameter quartz tube, and placed at the center of an alumina chamber. First, 2 g sulfur powder (99.95%, Gouden) was loaded in a separate ceramic crucible and placed in the furnace upstream at the edge of the chamber. The distance between the center of the two crucibles was fixed at 20.5 cm. Prior to the growth, the furnace was first purged with a consistent flow rate of 400 sccm high-purity N for 20 minutes to remove any moisture and impurities. The deposition temperature and duration were varied to be 650 °C, 750 °C and 850 °C at 15 min, 30 min and 60 min, respectively. The furnace temperature was gradually increased from room temperature to 500 °C in 30 min before ramping up to the growth temperature by 10 °C min^−1^. N flow was maintained at 200 sccm during the heating and growth time. The furnace temperature was then cooled down naturally to 300 °C and then rapidly to room temperature by opening the furnace hood.

### Characterization of materials and devices

The thickness of the as-synthesized Mo and MoS_2_ thin film was determined by X-ray reflectivity (XRR) and recorded using Rigaku's SmartLab multipurpose diffractometer. The X-ray diffraction (XRD) pattern was recorded using the same diffractometer. The surface morphology was confirmed using a FESEM (FEI Quanta 400F) equipped with an energy-dispersive X-ray spectrometer (EDX; Oxford ICNA 400). Raman spectroscopy measurement was performed to confirm the formation and quality of MoS_2_ using a Renishaw confocal Raman spectrometer equipped with an inVia microscope with a motorized stage. A laser with 514 nm excitation wavelength and 1.0 μm spot size was used. The signal was collected through a 50× objective lens at room temperature. Photoluminescence (PL) spectroscopy was conducted using the same equipped instrument at 325 nm excitation wavelength. A Hall measurement system (Dexing Magnet) was used to measure the electrical properties by the van der Pauw method. The optoelectronic performance of the n-MoS_2_/p-GaN PD was tested using a Keithley source meter 2410, at different light power intensities. A 355 nm UV-LED was applied as the illumination source with an adjustable light power intensity from 51 μW cm^−2^ to 2.47 mW cm^−2^, calibrated using a THORLABS optical power meter. The transient response was characterized under zero bias and potential using a Yokogawa DLM2054 oscilloscope, and a Stanford Research synthesized function generator (Model DS345).

### Device fabrication

n-MoS_2_ was initially deposited on a p-GaN substrate using a shadow mask to allow the deposition of a position-selective film. Then, the n-MoS_2_/p-GaN heterojunction device was fabricated by making Ni/Au (5/70 nm) and comb-shaped Cr/Au (5/70 nm) contacts on p-GaN and n-MoS_2_ respectively using a shadow mask, which were then deposited by E-beam evaporation. The comb-shaped electrode on the n-MoS_2_ film has a finger width of 0.1 mm, a gap between fingers of 0.2 mm and a finger length of 2 mm. The fabricated devices were annealed at 300 °C for 1 h in an Ar atmosphere to improve the ohmic contact quality.

## Results and discussion

**Fig. 1:**
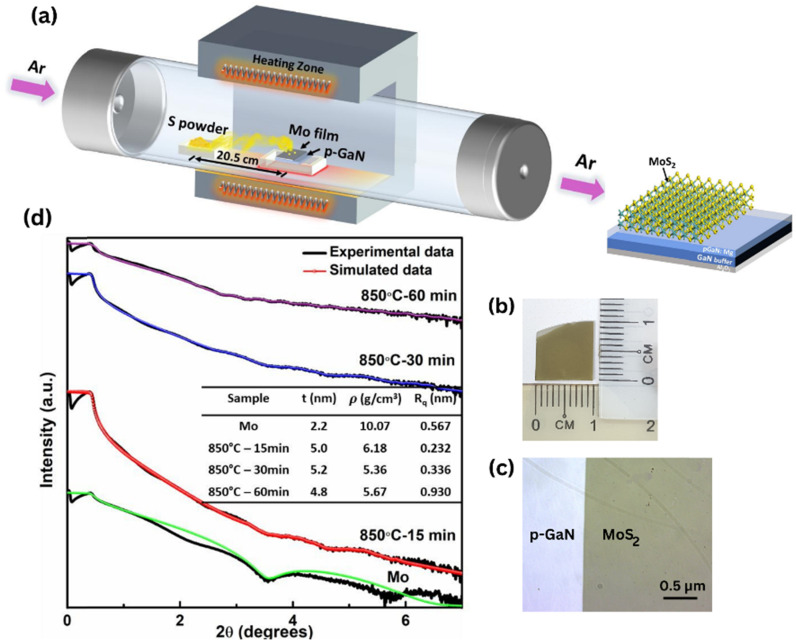
(a) Schematic illustration of the CVD setup. (b) Photograph image of the MoS_2_ film deposited on 1 cm × 1 cm p-GaN. (c) Optical image of the large-area patterned MoS_2_ film grown on p-GaN through a shadow mask, taken at 20× magnification. (d) X-ray reflectivity curve of the as-grown Mo and MoS_2_ films deposited on p-GaN at 850 °C – 15 min, 850 °C – 30 min and 850 °C – 60 min.

The synthesis of FL-MoS_2_ was carried out in a two-step process, as described in the experimental section and illustrated in Fig. 1a. Fig. 1b and c depict the photograph image and optical image of uniform and large-scale growth of MoS_2_ films successfully deposited on p-GaN respectively. The thicknesses of the as-deposited 2 nm Mo film and subsequent as-grown MoS_2_ thin films were confirmed by X-ray reflectivity (XRR) diffractogram, as illustrated in Fig. 1d. Raman vibrational modes are polarization dependent, where the polarization occurs along the in-plane and out-of-plane directions in the E^1^_2g_ (symmetric) and A_1g_ (anti-symmetric) modes, respectively. The resulting peak parameters (Δ*k*, FWHM, E^1^_2g_/A_1g_ intensity ratio) extracted from the Raman spectra of the films sulfurized at all parameters are summarized in ESI Table S1.† The frequency spacing of the two MoS_2_ vibrational phonon modes is generally a suitable quantity to indicate the number of MoS_2_ layers.^24^ The two modes have a frequency difference Δ*k* of 24.5–24.9 cm^−1^, for all sulfurization parameters corresponding to ∼6 to 7 layers of MoS_2_. We believe that the sulfur-Mo reaction has spread deeper into the Mo layer. This result confirms the thickness of MoS_2_ from the simulated XRR data analysis (Fig. 1d) of about 4.8–5.2 nm, where the thickness of a single MoS_2_ layer based on the previous report is ∼0.72 nm.^25^ The surface roughness averaged over the substrate can also be obtained from XRR measurements. XRR reveals the uniformity of the film, with surface roughness values of 0.232 nm, 0.336 nm and 0.930 nm sulfurized at 850 °C for 15, 30 and 60 minutes respectively, comparable to the literature values.^21,26^

**Fig. 2:**
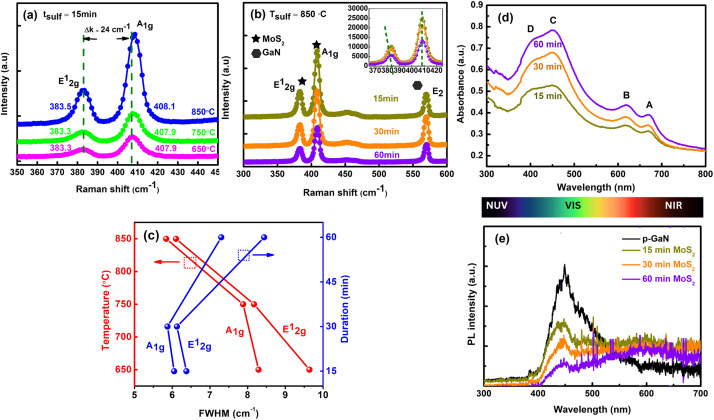
(a) Raman spectra (514 nm laser) of MoS_2_ films grown at different temperatures of 650 °C, 750 °C, and 850 °C for 15 min. (b) Raman spectra of 850 °C samples grown on p-GaN for different durations. (c) Full width at half maximum (FWHM) value of out-of-plane, A_1g_ and in-plane, E^1^_2g_ modes with respect to temperature and duration. (d) UV-Vis spectra of 850 °C samples grown on p-GaN for different durations. (e) PL measurements of 850 °C samples grown on p-GaN for different durations using a 325 nm excitation laser.

Raman spectra of MoS_2_ films sulfurized at different temperatures of *T*_sulf_ = 650 °C, 750 °C and 850 °C for 15 minutes are displayed in Fig. 2a. It is known that the quality of the deposited MoS_2_ layers can be examined from the Raman E^1^_2g_ and A_1g_ mode intensity and its linewidth. At *T*_sulf_ = 650 °C, the MoS_2_ Raman profile exhibits an asymmetric broadening of the peaks and is getting narrower as the deposition temperature increases to 850 °C. At a constant temperature of 850 °C and varying duration (Fig. 2b), MoS_2_ sulfurized for 15 and 30 minutes shows a comparable narrow linewidth and intensity. We believe that the change in linewidth is due to improved layer crystallinity in terms of the MoS_2_ crystallites size. To get a simplified picture of the quality, the full width at half maximum (FWHM) plot of E^1^_2g_ and A_1g_ modes with respect to the temperature (at *t*_sulf_ = 15 minutes) and duration (at *T*_sulf_ = 850 °C) was calculated and summarized in Fig. 2c. The E^1^_2g_ FWHM decreased from 9.64 to 6.10 cm^−1^ and A_1g_ FWHM decreased from 8.29 to 5.84 cm^−1^ as the temperature increased from 650 to 850 °C. Our results are comparable to the FWHM value obtained by Shahzad *et al.*^22^ When the duration is increased to 60 minutes at a constant 850 °C temperature, the FWHM increases dramatically. Several factors that contribute to the broadening of Raman modes are poor crystallinity, crystal defects, and grain size of the film crystal structure^27^ and the higher degree of structural order in both the in- and out-of-plane directions of the MoS_2_ films,^19,26^ which will be discussed further in the XRD and FE-SEM section.

The absorption spectra for the FL MoS_2_ nanosheets prepared at 850 °C for different sulfurization durations are displayed in Fig. 2d. The excitonic peaks arising from the Brillouin zone's *K* point are clearly visible at 668 ± 1 (A) and 616 ± 1 nm (B).^28^ The direct transition from the deep valence band (VB) to the conduction band (CB) might be assigned to the thresholds at 449 ± 1 (C) and 391 ± 1 nm (D).^29^

Fig. 2e illustrates the respective photoluminescence (PL) measurements for the three samples, conducted to explore the optical behaviour of the two interfaces. The spectra of p-GaN and sulfurized MoS_2_/p-GaN at various annealing time points are arranged together for comparison. The strong p-GaN peak at 447 nm suggests near-bandgap edge (NBE) emission, confirming that as-grown GaN has a high crystallinity and good p-type properties. The MoS_2_/GaN heterostructure's PL spectrum exhibits a minor blue shift at 442 nm and a broad peak from 546 nm to 669 nm, which could be attributed to MoS_2_. Furthermore, because the deposited MoS_2_ is approximately 6–7 layers, the PL characteristic peak strength corresponding to the A and B excitonic peaks (as indicated in Fig. 2d) is hardly visible and somewhat decreases as the sulfurization duration decreases from 15 minutes to 60 minutes.

**Fig. 3:**
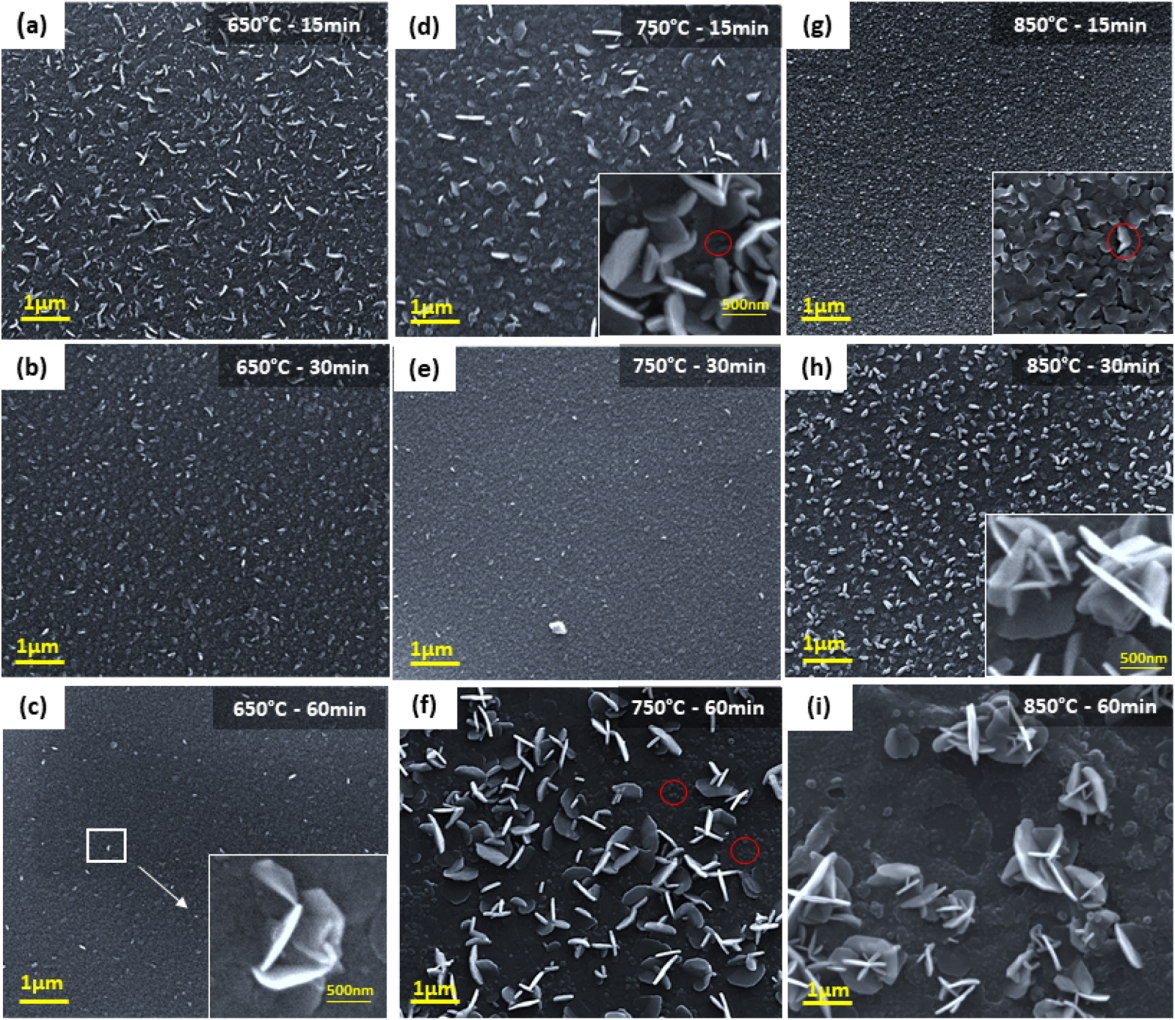
FE-SEM (a–i) images of MoS_2_ films grown on p-GaN at different sulfurization temperatures for different durations. The scale bar on the FE-SEM image is 1.0 μm. The inset shows the in-depth magnification image of (c) individual MoS_2_ growing in an out-of-plane manner to the horizontally aligned (HA) MoS_2_ layers (d) unaligned, bent, and overlapping vertically standing MoS_2_. The marked area shows few bent and overlapping horizontal MoS_2_ (g) horizontally aligned and bent MoS_2_ structures overlapping each other. The scale bars on the high-magnification FE-SEM images are 500 nm.

To gain a better understanding of the discussed sulfurization condition effects, FESEM observations were performed at intermediate states of MoS_2_ to understand the general network formations of synthesized MoS_2_. At the initial growth temperature and duration of 650 °C and 15 minutes, FESEM imaging reveals the growing horizontal and vertical structures as well as the extensive coverage of MoS_2_ on the p-GaN substrate (Fig. 3a). According to Vangelista *et al.* findings, at 500 °C, the pre-existing MoO_*x*_ film (derived from Mo precursor) has begun to synthesize into MoS_2_ (ref. 26) and the entire pre-existing MoO_*x*_ film serves as a seed for the MoS_2_ growth. The presence of vertical/horizontal oriented layers on the surface is due to insufficient energy to form a perfect 2D structure.

The film surface morphology shows denser and grainy features when the sulfurization duration was increased to 30 minutes, which became more apparent when the duration was increased to 60 minutes (Fig. 3b and c). Interestingly, in-depth magnification image shown in the inset in Fig. 3c reveals individual, vertically standing MoS_2_ nanosheets grown from the base of horizontal MoS_2_ layers. When the temperature was raised to 750 °C, significant vertically oriented films started to form (Fig. 3d).

Furthermore, we also observed overlapping and bent horizontal 2D layers grown parallel to the substrate (marked by the red circle of inset image Fig. 3d). The numerous 2D layers formed a coarse texture during which the duration was extended to 30 minutes (Fig. 3e). With the sulfurization duration continued to 60 minutes, the film was predominantly vertically oriented. Fig. 3f displays the clear image of edge-oriented MoS_2_ growing perpendicular to the basal planes. Additionally, there are a few spots on the substrate suffering from sulfur (S) depletion. However, incomplete sulfurization is unlikely due to the sulfur-rich environment supplied (∼2 g of S). We believe the sulfur depletion is caused by S desorption during long periods of annealing. Increasing the sulfurization temperature to 850 °C for 15 minutes results in significant grain growth (Fig. 3g). The high-resolution FE-SEM image displayed in the inset in Fig. 3g shows interconnected and densely grown, horizontally aligned (HA) and bent MoS_2_ structures overlapping each other. Few spots are observed to grow vertically standing on the GaN substrate (red circle). These differences indicate that the basal planes of the slightly increased and crystallographically improved grains of the MoS_2_ film began to align with the substrate surface. To note, Zeng *et al.* reported the majority of horizontal and few vertically grown bulk WS_2_ in their study as well.^30^ Interestingly, when the duration is extended to 30 minutes, we observed less coarse structures and highly dense vertically aligned (VA) MoS_2_ with regions of horizontally grown MoS_2_ films along the substrate's plane (Fig. 3h). The inset image shows that most of the edge-oriented structures are orthogonally aligned to the substrate surface, similar to what Deokar *et al.* achieved in their bulk MoS_2_.^31^ A longer sulfurization duration of 60 minutes results in a significant breakout of the MoS_2_ growth seen on the substrate plane, as displayed in Fig. 3i due to increased S desorption.

Jung *et al.* reveals that a thickness of more than 3 nm of metal seed layer leads to vertically oriented MoS_2_ perpendicular to the basal plane. They also stated that discontinuous 1 nm metal seed layers tend to grow into large areas of 2D horizontal films^32^ and that the mixed state of vertical and horizontal growth can occur in seed layers of intermediate thickness, as shown in our case. Sojková, *et al.*^33^ second the idea. On top of that, they also discovered that at 3 nm Mo thickness, the mixed state with combined VA and HA is achieved by decreasing the heating rate to 5 °C min^−1^ and is entirely HA at 0.5 °C min^−1^. However, it is worth noting that Sojková *et al.* attained the mixed state orientation when the S powder and Mo seed film are placed close to each other in the centre of the one-zone furnace, which differs from our growth mechanism. Hence, we presume that the mixed state of VA and HA observed in our grown MoS_2_ is due solely to the initial Mo seed thickness of 2 nm.

During the sulfurization process, nitrogen gas transports sulfur vapours, which diffuse into the MoO_*x*_ film and get converted into sulfide. The horizontally grown 2D films preferentially expose basal planes with low surface energy, in contrast to the vertically grown film, due to unconstrained vertical free volume expansion. The high surface energy on the exposed edges is compensated by vertically expanding and releasing the strain energy. In the growth of both vertical and horizontal layers, the horizontally grown 2D layers become discontinuous, bent, and overlapped, in this case, forming polycrystalline structures that release strain energy, as evidently in Fig. 3g. At a longer duration, chemical conversion occurs much faster than sulfur gas diffusion into the film, making sulfur diffusion the rate-limiting process. Diffusion along the layers *via* van der Waals gaps is expected to be much faster than the diffusion across the layers due to the anisotropic structure of MoS_2_. As a result, few MoS_2_ layers naturally orient perpendicular to the film, exposing van der Waals gaps and allowing for quick reaction. Additionally, sulfur desorption is also believed to occur as a result of the slow diffusion across the layer.

Moreover, Li *et al.* emphasize the significance of choosing a specific substrate for MoS_2_ growth to improve alignment due to the facet-dependency of the growth orientation.^34^ Hence, we believe that VA and HA MoS_2_ obtained in our study is due primarily to the GaN substrate effect.^35,36^

**Fig. 4:**
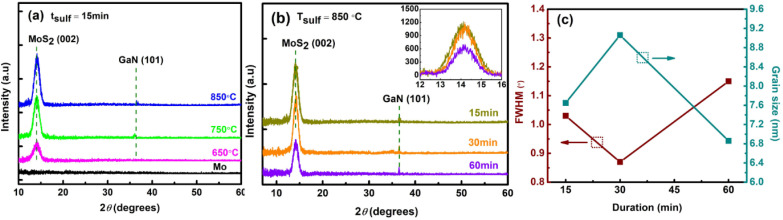
(a) HR-XRD spectra of the MoS_2_ films grown at different temperatures of 650 °C, 750 °C, and 850 °C for 15 min. (b) HR-XRD patterns of the 850 °C sample grown for different durations. (c) Full width at half maximum (FWHM) of the main XRD peak and the corresponding grain sizes of 850 °C sample as a function of sulfurization duration.

The crystalline structure and composition of the MoS_2_ films were further investigated by X-ray diffraction (XRD). The HR-XRD diffractogram of MoS_2_ sulfurized at *T*_sulf_ of 650 °C, 750 °C, and 850 °C for 15 minutes is shown in Fig. 4a. The as-deposited Mo film showed no diffraction peak, whereas the sulfurized film produced a main diffraction peak at ∼14.2° related to the (002) plane for the 2H-phase of MoS_2_ and a distinct peak at 2*θ* ≅ 36.8° corresponding to the (101) plane of GaN (PDF no 01-074-0243). The relatively high peak intensity at the (002) plane confirms that the MoS_2_ film grows preferentially with high crystallinity along the *c*-axis. Aside from that, it is worth noting that we were able to synthesize crystalline MoS_2_ from an amorphous Mo precursor.

The quality of the MoS_2_ deposited at all parameters was determined from the FWHM of its (002) peak and is presented in ESI Table S2.† As the sulfurization temperature increases over a constant duration of 15 and 30 minutes, the diffraction peaks become sharper (FWHM decreases), implying that the crystalline quality is improved. Nevertheless, a different trend is noticed when MoS_2_ is sulfurized for a constant duration of 60 minutes, in which the FWHM follows the order of 650 °C > 850 °C > 750 °C. A similar pattern is observed when the duration is prolonged, in which the quality is improved as the duration increases. However, at temperatures of 750 °C and 850 °C, the quality of sulfurized MoS_2_ drops from 30 → 60 → 15 minutes and 30 → 15 → 60 minutes respectively. To note, the sudden quality drop for the 850 °C – 60 minutes sample is due to the S desorption, as shown in Fig. 3i.

The EDX spectrum corresponding to the FE-SEM image for the film sulfurized at 850 °C for 15, 30, and 60 minutes (see ESI Fig. S3†) demonstrates the presence of elements Mo and S in which the atomic percentage ratio of Mo/S is ∼1 : 1.82, 1 : 1.91, 1 : 1.43 for the film sulfurized at 850 °C for 15, 30, and 60 minutes. Upon prolonged sulfurization to 30 minutes, the stoichiometry improves nearly to the stoichiometry ideal value. The protracted sulfurization to 60 minutes, however, results in a drop in the Mo/S ratio. This indicates that 30% or more of sulphur sites are vacant. Surprisingly, there is a significant increase in the atomic percentage of O_2_ observed in the 60 minutes sample. The defects present in the materials such as chalcogen vacancies in layered metal chalcogenides can serve as favourable sites for O_2_/H_2_O adsorption. This behaviour would trigger the breakdown of 2D materials, which could behave as detrimental active traps in working electronics^37^ and causes additional scattering of the carriers.

Fig. 4b shows the XRD plot of the high-intensity peak of MoS_2_ sulfurized at a fixed temperature of 850 °C for 15 to 60 minutes. As expected, the XRD peaks for the MoS_2_ sample grown at *t*_sulf_ = 15 minutes and 30 minutes show the highest comparable intensity peaks with a narrow width implying excellent crystalline quality. Furthermore, no peaks were shifted along the (002) plane in the inset in Fig. 4b, indicating that thin MoS_2_ films do not experience significant compressive stress during the deposition process.^38,39^ The average crystallite size of MoS_2_ can be determined from the broadening of the (002) diffraction peaks using Scherrer's formula:^40^1
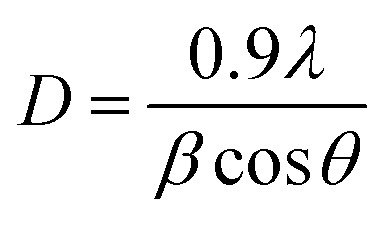
where *λ* is the X-ray wavelength, *β* is the broadening diffraction at half height of (002), and *θ* is the Bragg angle.

Fig. 4c shows the plot of the FWHM of the MoS_2_ (002) peak and the corresponding grain sizes of MoS_2_ synthesized at 850 °C for 15, 30 and 60 minutes. The high FWHM of 15 and 60 minutes sample reflects the small crystallite size. As the crystallite size is greatly reduced, the grain boundary area increases. This fact may reduce the MoS_2_ electrical conductivity [10], which will be explored further in the device photodetector (PD) performance section. In addition, we note the increment in the crystallite size as the temperature rises. Thermal energy from the process temperature influences film crystallization, and a sufficient supply of thermal energy increases the MoS_2_ grain size.^43^

According to Kong *et al.*, an edge-terminated structure has a smaller E^1^_2g_ peak intensity of about 30% than an A_1g_ peak intensity.^41^ This is comparable to the results we obtained (see Table S1†) for the E^1^_2g_/A_1g_ Raman peak intensity of ∼40%, which explains the presence of horizontally/vertically oriented structures. The sulfurized MoS_2_ film at a higher temperature and longer duration also has better crystalline quality, as evidenced by a decrease in peak widths in the Raman spectrum and XRD diffractogram (decrease FWHM). To note, the S desorption shown in Fig. 3f and i reflects the abrupt quality drops in XRD analysis.

**Fig. 5:**
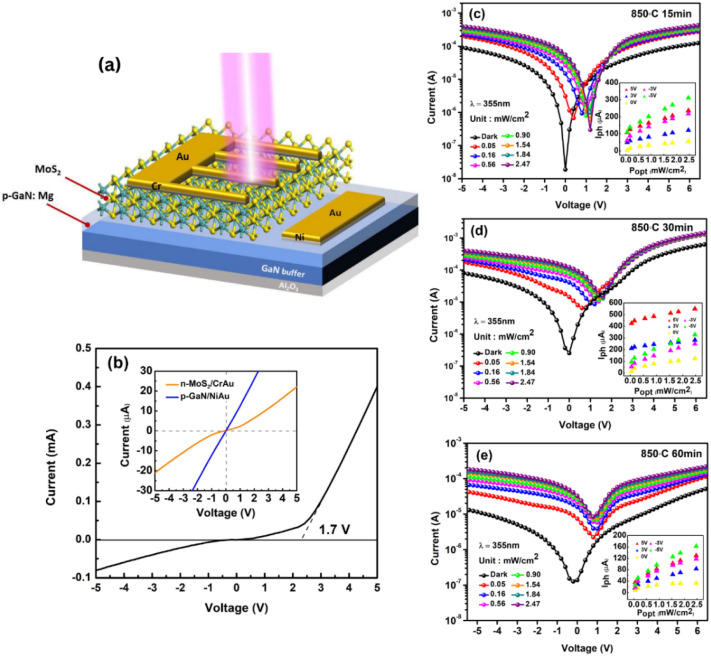
(a) Schematic diagram of the n-MoS_2_/p-GaN heterojunction photodetector. (b) Dark *I*–*V* curve of the n-MoS_2_/p-GaN heterojunction photodetector and the metal–semiconductor contact properties (inset). (c–e) Photoresponse of the n-MoS_2_/p-GaN heterojunction PD under varying light intensities. Inset shows the light intensity dependence of the photocurrent at different voltage biases.

Although vertically oriented TMDC layers offer great performances as an electrochemical HER catalyst, many published studies have demonstrated the excellent performance of vertically oriented MoS_2_ as a photodetector.^30,42–44^ In this work, we focus on examining the performances of deposited 2D MoS_2_ on the p-GaN substrate as a 2D/3D photodetector (PD) and correlate it with our characterization data. Samples sulfurized at 850 °C for 15, 30, and 60 minutes were used as the active layer to construct an n-MoS_2_/p-GaN heterostructure, as shown in Fig. 5a. The 15 and 30 minutes samples are selected for their highest quality, while the 60 minutes sample is selected to confirm the PD performance. Fig. 5b shows the dark *I*–*V* curve of the n-MoS_2_/p-GaN heterojunction photodetector (dark current of the 30 minutes device is taken for illustration purposes) and a clear rectifying characteristic with a small threshold voltage of ∼1.7 V, which is lower than the reported threshold voltage by Hyun *et al.*^45^ The quasi-linear *I*–*V* relationship shown in the inset in Fig. 5b indicates that both the Ni/Au electrodes on the p-GaN film and the Cr/Au electrode on the n-MoS_2_ film have good ohmic contact. As a result, the excellent rectifying characteristic is due to the formation of p–n heterojunctions between the GaN and MoS_2_ layers. This rectification characteristic demonstrates the presence of the built-in electric field.

Room-temperature MoS_2_ Hall effect studies were first performed to study the electrical properties. To avoid the influence of p-GaN, the measurement was done using MoS_2_ grown directly on the sapphire substrate. The results indicate that the deposited MoS_2_ samples have n-type carriers. The electron concentration and mobility of the MoS_2_ layer were estimated to be 9.58 × 10^13^ cm^−3^, 7.9 cm^2^ V^−1^ s^−1^; 1.70 × 10^14^ cm^−3^, 16.5 cm^2^ V^−1^ s^−1^; and 5.29 × 10^13^ cm^−3^, 3.5 cm^2^ V^−1^ s^−1^ for 15, 30, and 60 minutes respectively. Detailed results can be found in ESI Table S3.†

After the characterization of the MoS_2_/GaN p–n photodetector in the darkness, the relationship between photocurrent and incident light intensity was investigated to thoroughly explore the optoelectronic properties and its response to external illumination. The n-MoS_2_/p-GaN heterojunction was illuminated at 355 nm with different light intensities ranging from 51 μW cm^−2^ to 2.47 mW cm^−2^, and the logarithm plot of the IV curve as a function of light intensity is plotted in Fig. 5c–e to show the trend comparison. Because of sufficient incident light absorption and increased photogenerated carriers, photocurrents increase steadily with the light intensity. Intriguingly, our logarithmic plot displays an outstanding photoresponse towards UV light with three distinctive diode regions: (I) reverse bias region, (II) ideal linear diode region and (III) current injection region.

Moreover, at 100% light intensity of 2.47 mW cm^−2^, our n-MoS_2_/p-GaN photodetectors demonstrate a significant current on/off (*I*_light_/*I*_darkness_) ratio of ∼10^3^ for both 15 and 30 minutes devices, and 10^2^ for 60 minutes device at a bias of 0 V, implying that our photodetector also works as a photovoltaic. The inset in Fig. 5c–e shows the photocurrent plot as a function of incident light power under various bias conditions of −5 V, −3 V, 0 V, 3 V and 5 V. The photocurrent increases with the increase in bias, which can be attributed to an increased electric field around the conductive channel, resulting in a higher collection of photogenerated charge carriers. The 30 minutes device shows the highest photocurrent value generated at each respective bias voltage.

**Fig. 6:**
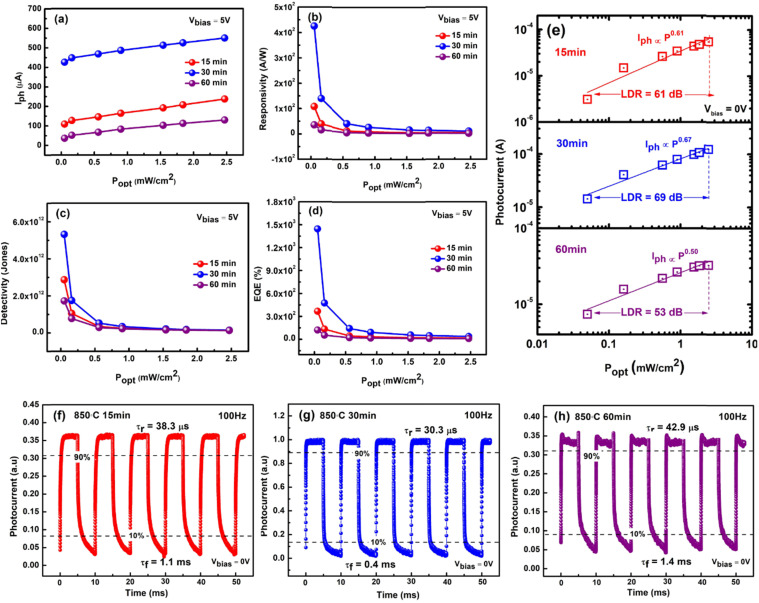
Plot of (a) net photocurrent *versus* light intensity. (b) Power-dependent responsivity, *R*. (c) Light density-dependent detectivity, D*. (d) External quantum efficiency (EQE) *versus* optical power density of the devices at 5 V bias voltage. (e) Logarithmic plot of photocurrent *versus* light intensity at 0 V bias voltage. (f–h) Photoresponse characteristics of the n-MoS_2_/p-GaN heterojunction to pulsed light irradiation (2.47 mW cm^−2^) at a frequency of 100 Hz under a voltage of 0 V and the estimated rise time (*τ*_r_) and fall time (*τ*_f_).

To quantitatively assess the overall properties of the heterostructure photodetector, the device performance was further analyzed by calculating the figure-of-merit (FOM) parameters such as responsivity (*R*), specific detectivity (*D*), external quantum efficiency (EQE) and linear dynamic range (LDR). The device sensitivity to incident light is denoted by *R* and calculated as eqn (2):2
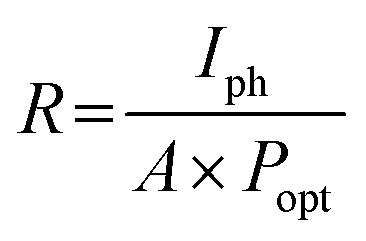
where *I*_ph_ is the net photocurrent obtained (subtracting dark current from the total current), *P*_opt_ is the incident light intensity and *A* is the total effective illuminated area of the device ∼0.2 mm^2^. The responsivity obtained from all devices at a bias voltage of 5 V is shown in Fig. 6b. At the lowest optical power of 51 μW cm^−2^, the 30 minutes PD reveals a massive maximum responsivity up to 4.25 × 10^2^ A W^−1^, 3.9 and 11.9 times higher than that of the 15 and 60 minutes PD, which have maximum responsivities of 1.08 × 10^2^ A W^−1^ and 35.6 A W^−1^ respectively (Fig. 6b). Aside from responsivity, the performance of a photodetector can also be expressed by detectivity (D*) defining the ability of a PD to detect weak signals. These two characteristic properties are related to each other according to eqn (3):3
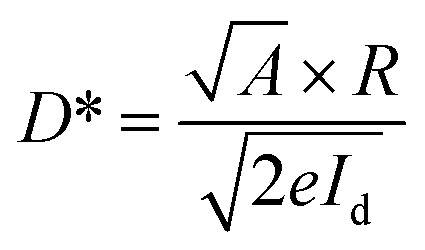


According to eqn (3), a high detectivity is attained at a low dark current and high responsivity. Fig. 6c depicts the highest peak detectivity of 5.31 × 10^12^ Jones for 30 minutes PD and is 1.8 and 3.0 times higher than that of the 15 and 60 minutes PD with the detectivity of 2.87 × 10^12^ Jones and 1.72 × 10^12^ Jones (1 Jones = 1 cm Hz^1/2^ W^−1^). Another essential parameter for the PD is external quantum efficiency (EQE), which calculates the ratio of photocurrent to incident photons using the responsivity value as written in eqn (4):4
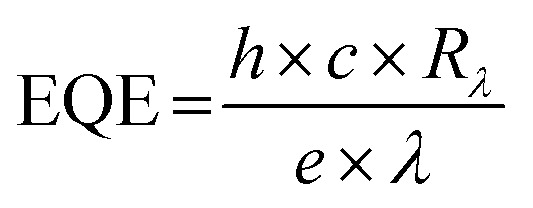
where *h*, *c*, *e* and *λ* are Planck's constant, the velocity of light, the unit charge and the excitation wavelength (355 nm) respectively. We observed the same trend of the EQE as in responsivity where the maximum EQE was calculated to be 3.6 × 10^2^%, 1.4 × 10^3^%, and 1.2 × 10^2^% for the 15, 30 and 60 minutes PD respectively (Fig. 6d). From all the parameters calculated, it is apparent that the 30 minutes PD exhibit the highest value of FOM.

The linear dynamic range (LDR), another figure of merit for PDs, displays the proportionate photocurrent dependent on the regulated power of the light sources. Linearity is critical for functional applications such as optical scanners and photometers. The LDR is theoretically defined as follows:5
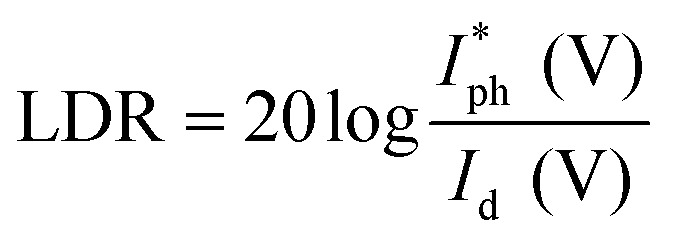
where 
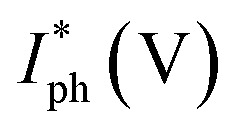
 is the maximal photocurrent density that maintains a linear relationship with light intensity. The LDR values measured under 1.84 mW cm^−2^ light illumination were calculated to be 61, 69, and 53 dB for 15, 30, and 60 minutes PD, at 0 V bias respectively (Fig. 6e). These values are on par with the reported photodetectors (≈42 dB, WS_2_/n-Si^46^ and ≈30 dB, Al_2_O_3_/MoS_2_ (ref. 47)), and some commercial photodetectors (such as GaN ≈ 50 dB (ref. 48) and InGaAs ≈ 66 (ref. 49)).

Time-resolved measurements were further investigated to assess the overall PD performance, which connects the MoS_2_/p-GaN PD, function generator, and oscilloscope. The frequency of the UV light was modulated by a function generator, while the photocurrent was measured and displayed as a function of time using an oscilloscope. The photoresponse characteristics of the PD were measured at a fixed frequency of 100 Hz using 355 nm UV light of 2.47 mW cm^−2^ intensity and a bias voltage of 0 V. The graph is presented in Fig. 6f–h. The rise time (*τ*_r_, the time interval from 10% to 90% of the maximum photocurrent) and fall time (*τ*_f_, the time interval from 90% to 10% of the maximum photocurrent) of a PD's response to an impulse signal are generally used to characterize its speed in the time domain. The 30 minutes device generates the fastest response time of *τ*_r_ = 30.3 μs and *τ*_f_ = 0.4 ms compared to 15 minutes PD with slightly longer response times of *τ*_r_ = 38.3 μs and *τ*_f_ = 1.1 ms and 60 minutes PD with *τ*_r_ = 42.9 μs and *τ*_f_ = 1.4 ms respectively.

The power dependence of the photocurrent in a photodetector provides crucial knowledge about the mechanism underlying photocurrent generation. The photocurrent measured at *V* = 0 V as a function of incident light power for the device's three states is represented in Fig. 6e in a log–log plot. The graph clearly reveals a non-linear relationship, indicating that the photocurrent and illumination power are attributed to a power law. This relationship can be expressed as

6 *I*_ph_ = *AP*^*α*^

where *A* is a scaling constant and *α* is the dimensionless exponent of the power law. The value of the exponent *α* indicates the number of traps in the photodetecting system.^50^ In an ideal trap-free photodetector, *α* is equal to one (=1), indicating that the photocurrent scales linearly with illumination power and that the responsivity is constant as a function of power. When trap states (for minority carriers) are present in the system, *α* becomes smaller than 1 (<1) and the responsivity depends sub-linearly on the illumination power, effectively decreasing for higher illumination powers.^51^ Thus, the higher *α* means higher charge separation (lower trapping ratios), beneficial to achieve high-performance photodetectors.^52^ The power equation (straight lines) matches the experimental data (hollow squares) with exponent *α* being the highest for 30 min device (*α* = 0.67) and lowest for 60 min (*α* = 0.50) device. The value of exponent *α* is between 0.5 and 1, indicating a photoconductive (PC) dominated device. Intriguingly, the rapid response time attained by all three devices implies that the response time is unaffected by the interface traps.

The experimental findings reported above are summarized in Table 1 to provide a clear comparison on the devices. We see a decrease in both the dark current and the current under illumination after sulfurizing the MoS_2_/GaN p–n photodetectors for 60 minutes, as well as a decrease in reaction time. Statistically, the responsiveness and reaction time of all three photodetectors studied are significantly connected.

**Table 1:** Summary of the figure of merit of all three devices

Device	Responsivity (A W^−1^)	Detectivity (Jones)	EQE (%)	LDR (dB)	Rise time (μs)	Fall time (ms)
15 minute	108	2.87 × 10^12^	3.6 × 10^2^	53	38.3	1.1
30 minute	425	5.31 × 10^12^	1.4 × 10^3^	69	30.3	0.4
60 minute	35.6	1.72 × 10^12^	1.2 × 10^2^	61	42.9	1.4

Comparing the measured PD FOM of the three devices, the 30 minutes device evidently shows a large variation compared to the 15 and 60 minutes devices. Although the improved crystallography grain of the 15 minutes sample is expected to provide high responsivity, given that the majority of its configuration is parallel to the substrate, we believe the lacking of FOM obtained is due to stacking faults and other defects, which are undoubtedly present in the film with a number of grain boundaries, resulting in a high density of recombination centers for the photogenerated carriers and, inherently, a fractional power dependence of the photocurrent. The high FOM achieved by the 30 minutes device provides direct evidence of the performance-quality dependence.

**Fig. 7:**
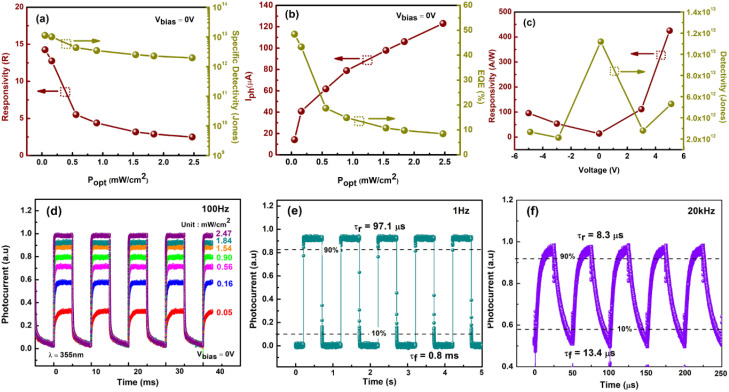
Light intensity dependence of (a) responsivity and specific detectivity and (b) photocurrent and EQE of the 30 minutes device at 0 V bias voltage. (c) Responsivity and specific detectivity under 51 μW cm^−2^ light illumination of 365 nm wavelength at varying bias voltages. Photoresponse of the 30 minutes n-MoS_2_/p-GaN heterojunction (d) by varying light intensities at a frequency of 100 Hz and under pulsed light irradiation (2.47 mW cm^−2^) at frequencies of (e) 1 Hz and (f) 20 kHz under a voltage of 0 V.

The state-of-the-art performance of our 30 minutes PD is elaborated further to compare with previously reported MoS_2_/3D-based PD and summarized in Table 2. At 51 μW cm^−2^ light intensity, and *V*_bias_ of 0 V, our PD possesses *R* and D* of 14.3 A W^−1^ and 1.12 × 10^13^ Jones respectively (Fig. 7a), higher than that of the reported PD by Zhuo *et al.* with *R* and D* of 0.187 A W^−1^ and 2.34 × 10^13^ Jones (ref. 53) confirming the MoS_2_ excellent performance as an active material in photodetector fabrication. In Fig. 7b, the calculated EQE could reach up to 48%. Additionally, it is worth noting that we managed to achieve the high *R*, D* and EQE at a low external bias voltage (0 V), in contrast to the relatively high bias voltage required by previous works. For instance, Zhang *et al.* attained R of 27.1 A W^−1^, D* of 1.70 × 10^10^ Jones and EQE of 92% at 5 V bias under 72 mW cm^−2^ of 365 nm light illumination.^54^ Competitively, at 5 V, our PD achieves a maximum *R* of 4.25 × 10^2^ A W^−1^, D* of 5.31 × 10^12^ Jones, and EQE of 1.44 × 10^3^% under extremely low 355 nm light intensity of 51 μW cm^−2^, as previously explained in detail (refer Fig. 6).

**Table 2:** Performance comparison of our n-MoS_2_/p-GaN PD with other reported MoS_2_/3D based PD

Device structure	MoS_2_ layers/growth method	Light source, *λ* (nm)	Self -powered	Responsivity (A W^−1^) at *V*_bias_	D* (Jones)	Response time (*τ*_r_/*τ*_f_)	Ref.
n-MoS_2_/p-GaN	FL/E-beam (Mo) + CVD (single zone)	355	Yes	14.3 at 0 V, 4.2 × 10^2^ at 5 V	1.12 × 10^13^, 5.31 × 10^12^	97.1 μs/0.8 ms (1 Hz), 38.3 μs/1.1 ms (100 Hz), 8.3/13.4 μs (20 kHz)	This work
MoS_2_/p-GaN	(NH_4_)_2_MoS_4_ + CVD (single zone)	265	Yes	0.187 at 0 V	2.34 × 10^13^	0.3/3.6 ms (100 Hz), 46/114 μs (5 kHz)	53
p-MoS_2_/n-2D GaN	Bulk/mechanical exfoliation	365	No	27.1 at 5 V	1.7 × 10^10^	300 ms/3.9 s	54
MoS_2_/n-GaN	FL/sputtering (Mo) + CVD (dual-zone)	365	No	∼10^3^ at 1 V	∼10^11^	∼5 ms	55
MoS_2_/GaN	Bulk/mechanical exfoliation	365	No	∼10^4^ at 1 V	7.46 × 10^12^	—	56
MoS_2_/un-GaN	Bulk/mechanical exfoliation	405	No	∼10^5^ at 5 V	∼10^14^	105.6/84.1 ms	17
p-GaN/SiO_2_/n-MoS_2_/graphene	1L/wet transfer CVD	633	Yes	10.4 at 0 V	1.1 × 10^10^	100 ms	57
GaN/h-bN/MoS_2_	1L/wet transfer CVD	400–700	—	1.2 mA W^−1^ at 9 V	—	500 ms	58

From the graph too, we can see that *R*, D* and EQE are inversely proportional to the light intensity, indicating the presence of minority carrier traps (holes) in the device, as explained in the previous power-dependent measurements. When the intensity of the illumination increases, fast rate of electron–hole pair separation occurs, resulting in a higher photocurrent. Photogenerated electrons in MoS_2_ are captured by trap states under low light intensity. Because of reduced recombination, the lifetime of photogenerated holes can be greatly extended, resulting in higher *R* and D*. However, as the light intensity increases, the available states decrease dramatically, eventually resulting in photoresponse saturation.^59^ The photocurrent's sub-linear behaviour suggests that trap states in the MoS_2_ layer or at the MoS_2_/p-GaN junction interface are to be accountable for this occurrence.

Fig. 7c shows a bias-dependent plot of *R* and *D*. The responsivity, *R*, behaves similarly to the photocurrent (refer inset Fig. 5), increases sharply with the increase in bias under both forward and reverse bias. The detectivity D*, however, is not monotonous, reaching a maximum at 0 V, decreases to ±3 V and increases back to ±5 V. Fig. 7d displays the photoresponse under varying *P*_opt_ at 0 V bias. The results indicate that when the UV light was turned on and off at 100 Hz frequency, the current alternately switched between high and low conductance with good consistency and repeatability. Notably, even when exposed to higher light intensities, the current increased only slightly. Given that the result was obtained at a high frequency of 100 Hz, we conclude that our n-MoS_2_/p-GaN PD can detect a very weak UV light, confirming that it is a self-powered UV PD. The time response of our PD was further modulated in the frequency range of 1 Hz to 20 kHz at a fixed *P*_opt_ of 2.47 mW cm^−2^. At 1 Hz frequency, the rise time (*τ*_r_) and fall time (*τ*_f_) are 97.1 μs and 0.8 ms respectively as demonstrated in Fig. 7e. At 20 kHz, our device obtained a remarkably fast response speed of *τ*_r_: 8.3 μs and *τ*_f_: 13.4 μs, indicating that the PD can follow rapidly changing UV light signals.

**Fig. 8:**
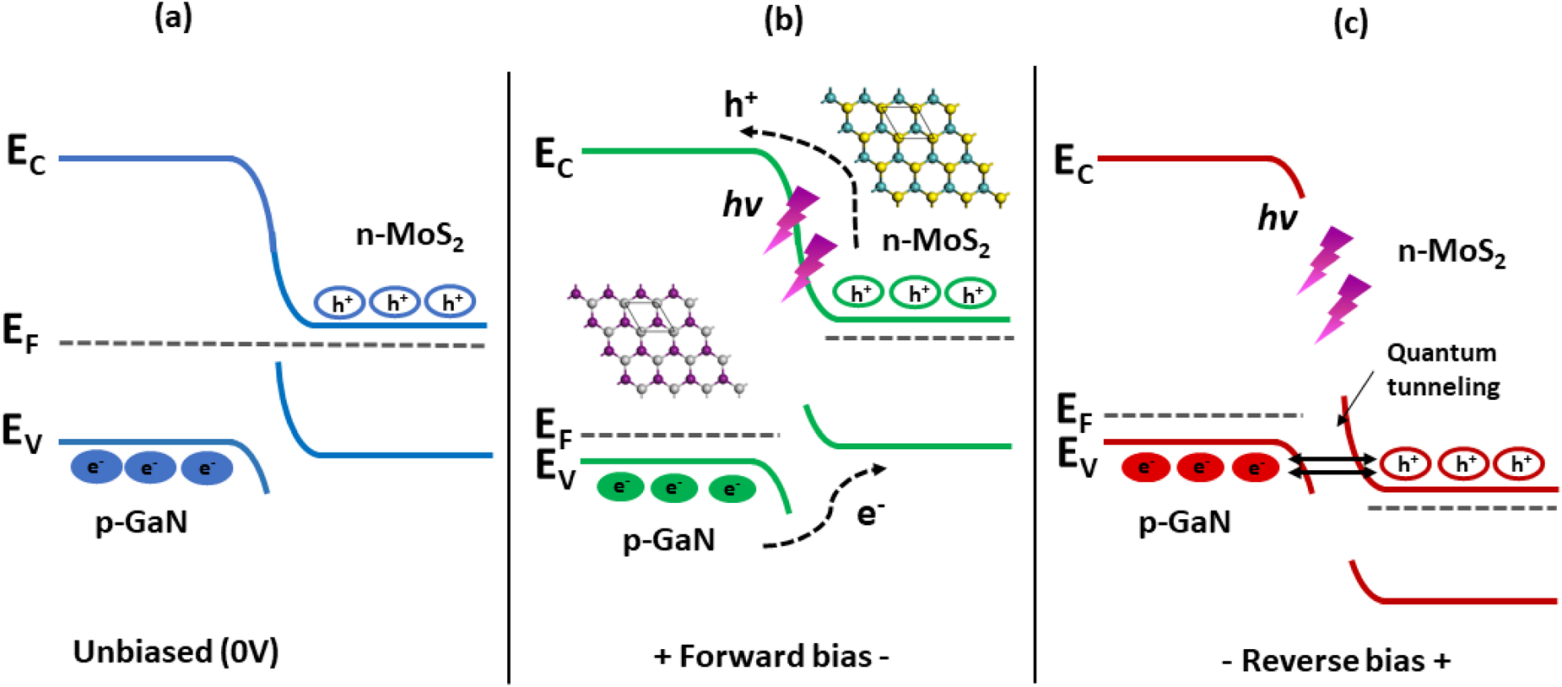
Energy band diagrams of the MoS_2_/GaN heterojunction under (a) 0 V bias and under UV illumination (b) at forward bias and (c) at reverse bias.

The energy band diagrams shown in Fig. 8 help to understand the entire electron–hole concept, which explains the enhanced photoresponse properties of GaN/MoS_2_ p–n. The bandgap (*E*_g_) of MoS_2_ and GaN is ∼1.42 eV (taken from the Tauc plot of MoS_2_ absorption spectrum in ESI, Fig. S4b†) and 3.4 eV, respectively. Exceptional heterojunctions can be formed due to close lattice match between MoS_2_ and GaN.^14^ Because of the difference in the Fermi level (*E*_F_), electrons in the MoS_2_ film will likely move to the GaN side once the p–n heterojunction between the MoS_2_ film and the p-GaN substrate is formed, whereas holes in GaN will tend to move to the MoS_2_ film. As a result, energy levels near the GaN surface will bend downward, while energy levels near the MoS_2_ surface will bend upward, and the Fermi levels of MoS_2_ and GaN will eventually align at the same level.^8,60^ This contributes to the built-in electric field near the MoS_2_/p-GaN interface.

Under UV light illumination (Fig. 8b), the absorption of the incident light generates more electron–hole pairs, which are quickly separated by the built-in electric field and transferred to the electrodes, giving rise to photocurrent, resulting in a rapid response time. The built-in electric field at the junction interface confirms that the device can be operated at zero bias. When a reverse bias is applied, the *E*_f_ (p-GaN) is raised to higher values, as shown in Fig. 8c. This gradually increases the electric field across the depletion region, resulting in the expansion of barrier potential. This will allow many accessible states for the hole and electrons to tunnel into the GaN and MoS_2_ region respectively. Under light illumination, electron–hole pairs are generated, which are then separated by this large barrier potential and collected by the electrodes. Furthermore, regardless of the atomic thickness, MoS_2_ can strongly interact with incident light while maintaining high transparency^61,62^ and the naturally passivated surfaces can protect the devices from surface leakage current, which is a critical issue that must be addressed rigorously in semiconductor film processing technologies.^63^

## Conclusion

We have successfully grown high-quality, large-area and flat-surface FL-MoS_2_ on p-GaN *via* a simplified sulfurization technique of an E-beam-deposited Mo seed layer by single-zone CVD. The controllable orientation of thin MoS_2_ layers at a 2 nm Mo seed layer was demonstrated by tuning the sulfurization parameters (temperature and duration). At 650 °C for 15 minutes, the sulfurized film is composed of low-crystallized and unaligned 2D structures. A temperature increase to 850 °C improves the structural quality and increases the crystallite size. A longer sulfurization time oriented the film to be more of the VA film, but annealing for 60 minutes caused S desorption, which degraded the film quality. The MoS_2_ film sulfurized at 850 °C for 30 minutes possesses the highest quality and crystallite size. The MoS_2_ film is composed of numerous MoS_2_ nanosheets, the majority of which are VA and only a few are HA to the p-GaN substrate. Additionally, we successfully deposited VA MoS_2_ on p-GaN by the newly MoS_2_ position-selectivity method directly on the p-GaN substrate and employed as a heterostructure for photodetector device application. Although the 850 °C 15 minutes device improved the crystallography grain predicted to have a high responsiveness, the lack of FOM obtained provides direct evidence of the performance-quality dependence. The 30 minutes device's high FOM yields a clear proof of the performance-quality correlation. The surface morphologies of the deposited MoS_2_ film contribute to the performance of the MoS_2_/p-GaN heterojunction photodetector. Light trapping phenomena and high responsivity from the textured nanostructures at the surface may have improved photo-carrier generation and collecting efficiency. Our PD exhibits the highest photoresponsivity, *R* of 425 A W^−1^, a specific detectivity, D* of 5.31 × 10^12^ Jones and EQE of ∼10^3^% for 355 nm excitation at 51 μW cm^−2^ under 5 V bias condition. Under 0 V bias, we achieved *R* of 14.3 A W^−1^, D* of 1.12 × 10^13^ Jones, and a high *I*_on/off_ ratio of ∼10^3^ confirming it as a self-powered device. The device also responds quickly to the UV signal of 8.3/13.4 μs (20 kHz).

Finally, we demonstrated that our V-MoS_2_/p-GaN works under reverse bias, making it a dual-functional PD. Even though horizontally grown 2D TMDC is known to be promising for optoelectronics due to high on/off ratios (>10^5^) as transistors, we have demonstrated that high-quality VA MoS_2_ grown under optimal conditions and on a particular substrate has quite outstanding performance as a photodetector. Such knowledge could further allow for the direct synthesis of novel 2D TMDC/3D heterostructures that consist of both vertically and horizontally grown 2D layers.

## Author contributions

N. A. A. Z. and R. Z. design the experiment. N. A. A. Z. conducted all experimental works, collected, and analysed the data and drafted the manuscript. R. Z. and S. M. S. supervised the project. N. H. Z. and A. H. A. R. participated in materials and device characterization. All authors reviewed the manuscript.

## Conflicts of interest

There are no conflicts to declare

## Supplementary Material

NA-005-D2NA00756H-s001
