# Supplementary material for: Sulfurization engineering of single-zone CVD vertical and horizontal MoS2 on p-GaN heterostructures for self-powered UV photodetectors
Source: Nanoscale Adv. 2023 Jan 10;5(3):879–92. doi: 10.1039/d2na00756h (PMC9890942; doi:10.1039/d2na00756h)
Supplement: NA-005-D2NA00756H-s001 [file NA-005-D2NA00756H-s001.pdf]

Electronic Supplementary Material (ESI) for Nanoscale Advances.
This journal is © The Royal Society of Chemistry 2022

Supporting Information

Sulfurization Engineering of Single Zone CVD vertical and horizontal MoS_2 on p-GaN heterostructure for self-powered UV PD

Nur 'Adnin Akmar Zulkifli[a], Nor Hilmi Zahir[b], Atiena Husna Abdullah Ripain[a], Suhana Mohd Said[c], Rozalina Zakaria[a,*]

[a]Photonic Research Centre, Institute for Advanced Studies, University Malaya, 50603 Kuala Lumpur, Malaysia

[b]Low Dimensional Material Research Center (LDMRC), Physics Dept. Faculty of Science, University Malaya, 50603 Kuala Lumpur, Malaysia

[c] Department of Electrical Engineering, University of Malaya, 50603 Kuala Lumpur, Malaysia

*Corresponding authors: rozalina@um.edu.my (R.Z.)

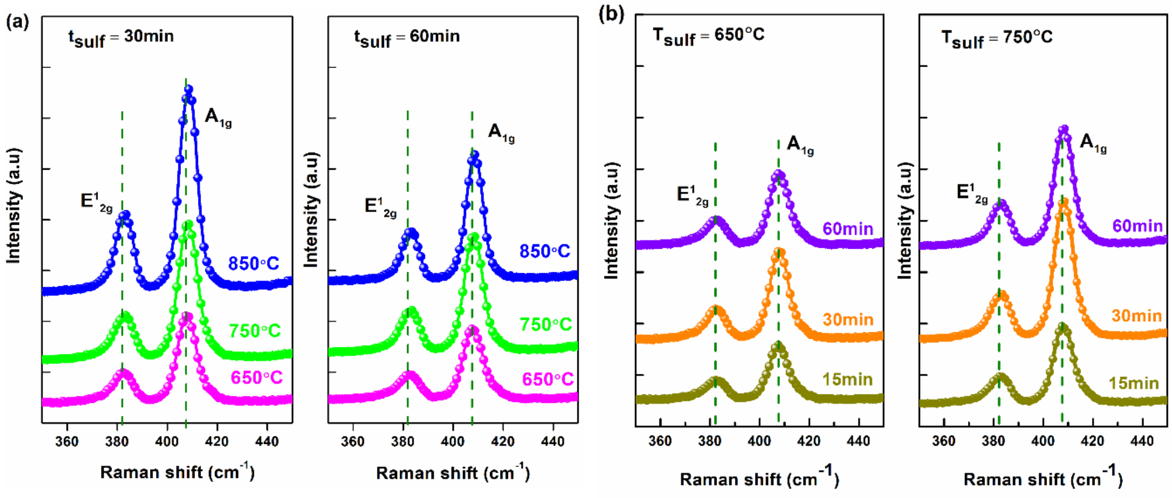

Figure S1. (a) Raman spectroscopy of MoS_2 sulfurized for 30- and 60 minutes at different growth temperatures. (b) Raman spectroscopy of MoS_2 grown at 650 °C and 750 °C with different sulfurization duration.

Table S1. Values of Raman E^1_{2g} and A^1g peaks position, FWHM, and E^1_2 / A^1g relative intensity ratio for grown MoS_2 at different sulfurization temperatures and duration

	15 min			30 min			60 min		
	**650 °C**	**750 °C**	**850 °C**	**650 °C**	**750 °C**	**850 °C**	**650 °C**	**750 °C**	**850 °C**
**E^1_{2g} peak FWHM (cm^{-1})**	9.64	8.17	6.10	8.75	6.92	5.90	8.60	7.12	7.75
**A_{1g} peak FWHM (cm^{-1})**	8.29	7.88	5.84	7.43	6.29	5.70	7.31	6.43	6.84
**E^1_{2g} peak position (cm^{-1})**	383.3	383.3	383.5	383.3	383.4	383.2	383.3	383.4	383.5
**A_{1g} peak position (cm^{-1})**	407.9	407.9	408.1	407.9	407.9	408.1	407.9	407.9	408.1
**Δk (cm^{-1})**	24.6	24.6	24.6	24.6	24.5	24.9	24.6	24.5	24.6
**E^1_{2g} peak intensity (a.u)**	3,056.88	3.787.28	10,980.60	4,043.23	5,592.29	7,682.77	3,470.98	5,126.09	5,646.40
**A_{1g} peak intensity (a.u)**	6,469.40	8,793.11	27,140.60	9,623.74	14,567.20	18,761.80	7,987.07	12,383.30	13,264.40
**E^1_{2g}/ A_{1g} intensity ratio**	0.47	0.44	0.40	0.42	0.38	0.41	0.43	0.41	0.43

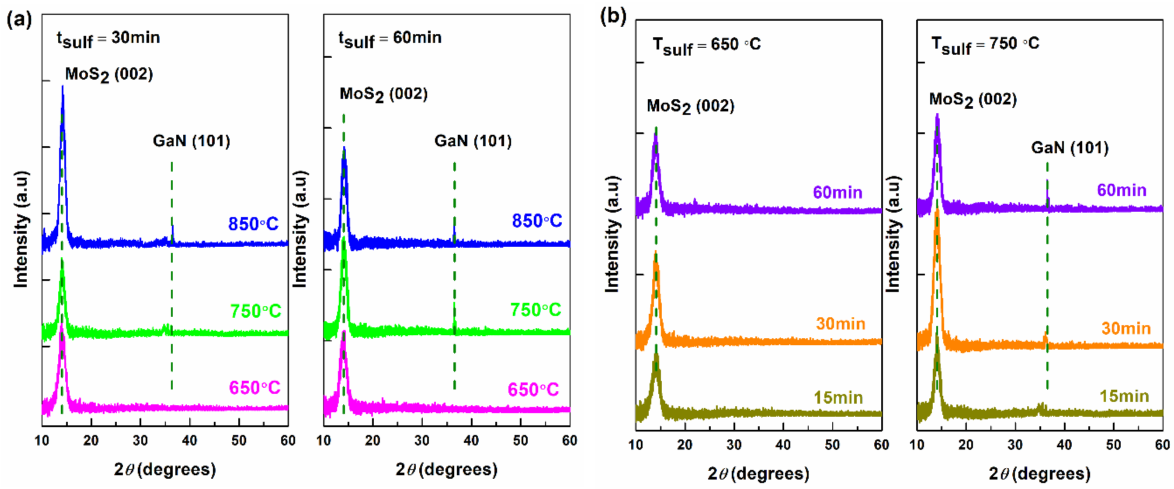

Figure S2. (a) XRD diffractogram of MoS_2 sulfurized for 30- and 60 minutes at different growth temperatures. b) XRD diffractogram of MoS_2 grown at 650 °C and 750 °C with different sulfurization duration.

Table S2. Data extracted from XRD measurements

	15 min			30 min			60 min		
	**650 °C**	**750 °C**	**850 °C**	**650 °C**	**750 °C**	**850 °C**	**650 °C**	**750 °C**	**850 °C**
**(002) peak FWHM (°)**	1.49	1.21	1.03	1.29	1.09	0.87	1.26	1.07	1.15
**Crystallite size (nm) (± 0.1)**	5.29	6.52	7.65	6.11	7.23	9.06	6.26	7.37	6.86

Table S3. The results of room-temperature Hall effect measurements of MoS_2 film sulfurized at 850°C for 15-, 30-, and 60 minutes.

MoS_2 Sample	Type	Carrier density (/cm^3)	Mobility (cm^2/V·s)
15 minutes	n	9.58×10^{13}	7.9
30 minutes	n	1.70×10^{14}	16.5
60 minutes	n	5.29×10^{13}	3.5

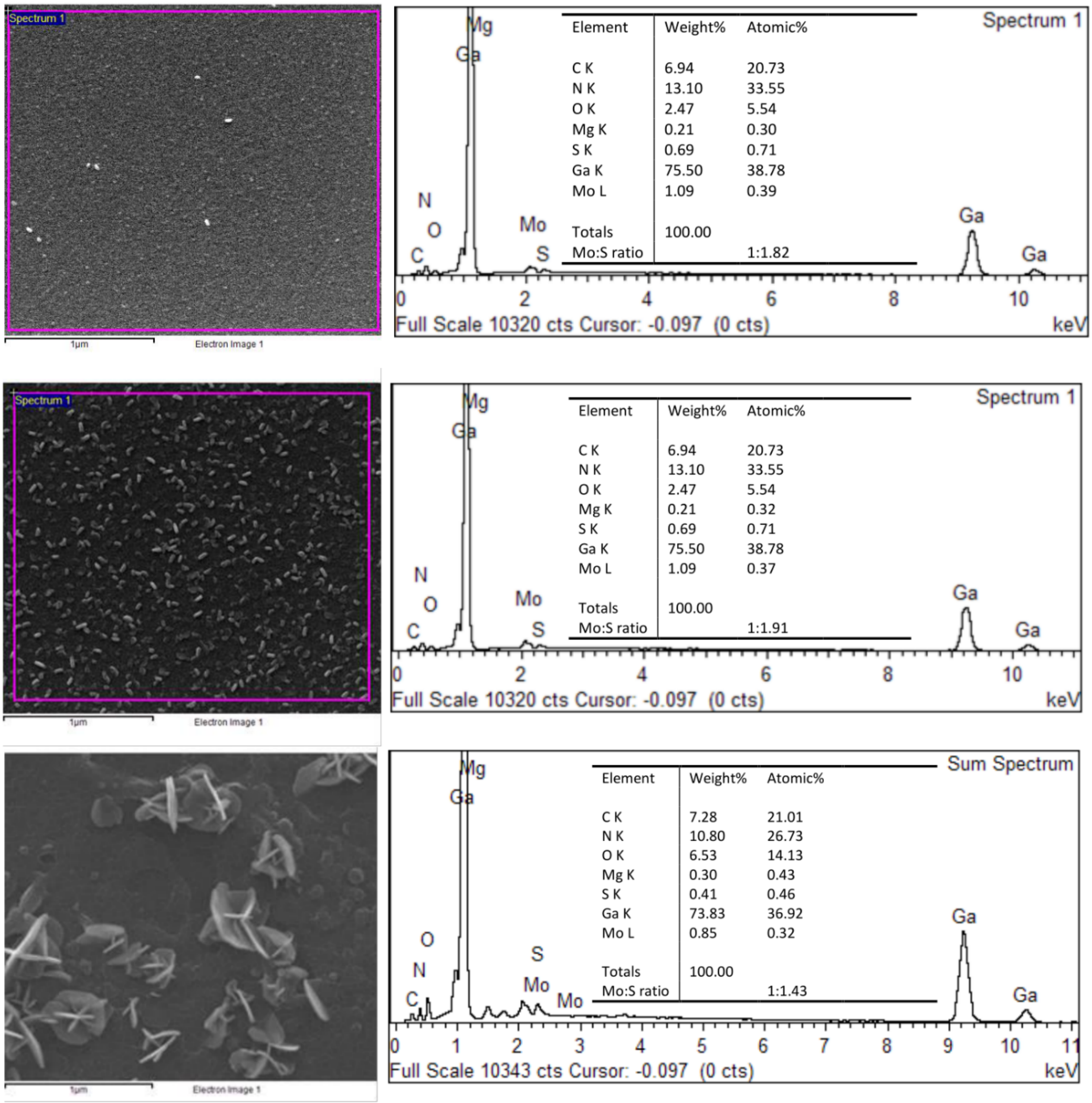

Figure S3. Overall EDX area analysis and elemental quantification of FE-SEM images of MoS_2 grown at conditions (a) 850°C – 15 minutes (b) 850°C – 30 minutes (c) 850°C – 60 minutes

Quantifying Mo and S elements is difficult since the emission energies of Mo Lα (2.292 keV) and S Kα (2.309 keV) are very close to each other. But the sum of Mo and S is reliable to quantify the amount of MoS_2. The EDX spectrum corresponding to the FE-SEM image reveals the presence of elements Mo and S in which the atomic content ratio Mo/S is ~1:1.82, 1:1.91, 1:1.43 for the film sulfurized at 850°C for 15-, 30-, and 60- minutes. Upon longer sulfurization to 30 minutes, the stoichiometry improves nearly to the stoichiometry ideal value. A prolonged sulfurization however results in dropped Mo/S ratio. This means about ~30% of sulfur sites are vacancies.

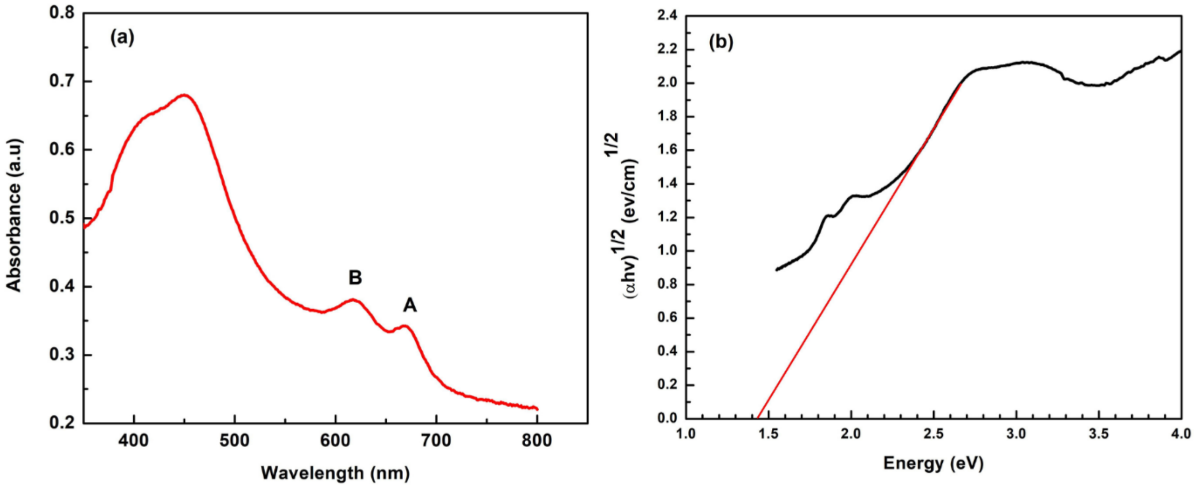

Figure S4. a) UV–vis spectrum showing the characteristic excitonic peaks of 2H-MoS_2 nanosheets b) Determination of absorption band gaps through absorbance spectra.

Tauc plot of $(\alpha hv)^{1/2}$ versus energy hv is shown in Figure S4 (b). By extrapolating the linear part of the curves to the energy axis (at $\alpha hv = 0$), we obtained the band gap energy of ~1.42 eV.

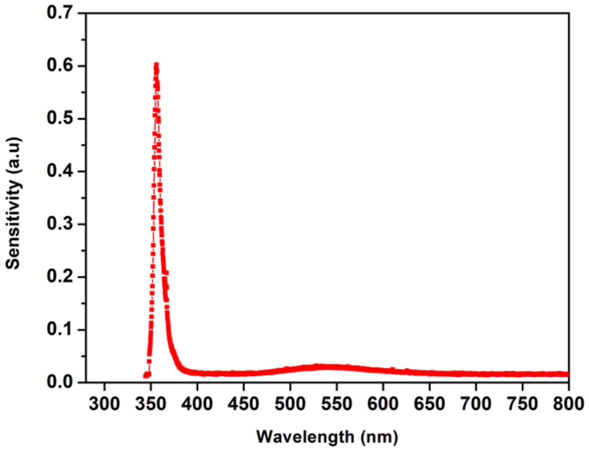

Figure S5. Response spectra of the 355 nm UV LED.
